# Can the humped animal's knee conceal its name? Commentary on: “The roles of shared vs. distinctive conceptual features in lexical access”

**DOI:** 10.3389/fpsyg.2015.00418

**Published:** 2015-04-10

**Authors:** Maria Montefinese, David Vinson

**Affiliations:** ^1^Department of Neuroscience, University of PadovaPadova, Italy; ^2^Department of Experimental Psychology, Institute for Multimodal Communication, University College LondonLondon, UK

**Keywords:** shared feature, distinctive feature, picture-word interference, lexical access, lexical selection by competition, response exclusion

The representation of meaning is a pivotal topic for theories of language processing. A prevalent view is based on semantic features, considering conceptual representations as distributed patterns of activity across sets of features related to different aspects of knowledge and experience (e.g., Rosch and Mervis, 1975; Vigliocco et al., 2004; Cree et al., 2006). These features can vary in their relative salience to a concept's meaning and co-occur to various degrees across concepts. For example, *distinctive* features occur in few concepts and allow people to distinguish very similar concepts (Grondin et al., 2009), while *shared* features occur across many concepts thus indicating similarity among them (Montefinese et al., 2014a). Existing studies yield conflicting results about the relevance of featural characteristics (Montefinese et al., 2014b), leaving it unclear what theoretical interpretations can be drawn.

Vieth et al. (2014) recently sought to clarify the role of feature distinctiveness in a picture-word interference (PWI) task. In Experiment 1, they employed categorically-related distractor-target pairs matched for semantic similarity, while manipulating distinctiveness of the distractor feature. Experiments 2 and 3 employed part-whole distractor pairs while manipulating distinctiveness and visibility of the distractor feature in the target picture. Distinctiveness had an extremely constrained effect: non-distinctive feature distractors slowed target naming, but only at an SOA of −150 ms and only when they were visible in the picture (Experiment 3). The authors conclude that semantic interference in the PWI paradigm is due to conceptual feature overlap and thus consistent with lexical selection by competition (Roelofs, 1992) rather than the response exclusion hypothesis introduced by Mahon et al. (2007). Unfortunately, these conclusions are undermined by lack of a crucial statistical interaction to motivate follow-up testing, poor control of semantic measures, and an inadequate account of the role distinctiveness would play in lexical retrieval.

Vieth et al. found one effect of distinctiveness: in Experiment 3, “non-distinctive part-whole target relations showed picture naming latencies significantly at −150 ms SOA compared to their matched unrelated pairings” (p. 9). However, such conclusions are not warranted by the evidence provided. The authors drew conclusions from partial interactions without a significant higher-order interaction. However, this is a common problem in studies employing factorial ANOVA (see Nieuwenhuis et al., 2011), and is likely to inflate the likelihood of Type I error particularly in repeated-measures ANOVA, which is anticonservative for designs including crossed random effects by-participants and by-items (Quené and van den Bergh, 2008). We therefore wonder whether the most appropriate conclusion from Experiment 3 is that, as in Experiments 1 and 2, feature distinctiveness does not affect the degree of interference in PWI.

Moreover, although the authors made careful efforts to match lexical variables between conditions, some crucial semantic variables remain uncontrolled. For example, there are substantial differences in dominance of the distinctive and non-distinctive features Vieth et al. used in their experiments. Moreover, hardly any of the non-distinctive features appear in McRae et al.'s (2005) norms (Table 1), indicating that participants do not find features like “*knees*” of CAMELS sufficiently salient to report them. Classic feature-verification studies using very similar item sets (e.g., Conrad, 1972; Glass et al., 1974) suggest that distinctiveness effects are substantially reduced or eliminated by taking dominance into account; and more recent work by O'Connor et al. (2009) suggests that non-distinctive features are much more highly associated with superordinate terms (e.g., “animal” or “mammal”) than the basic-level terms employed by Vieth et al. Therefore, if dominance is a measure of a feature's semantic proximity to the target concept label (and thus its level of competition for lexical selection under selection-by-competition accounts), the activation of target concepts by non-distinctive features would depend on their dominance. Features that are salient for multiple concepts would activate competing concepts and interfere with their naming, while those that are not salient for any concept would not. Examples of these two types of non-distinctive features are, respectively, “*bone*” and “*skin*,” which were listed for none and 16 of the 541 concepts of McRae et al.'s norms. In brief, distinctiveness alone would not explain how strongly a feature can activate one or more target concepts. But let us set aside statistical and methodological concerns about Experiment 3 and assume that the effect they describe is real interference for visible non-distinctive part distractors at −150 ms SOA only. The authors do not adequately describe the processes that might have caused this temporally-selective effect, instead discussing the three-way interaction (SOA × part-relation × distinctiveness) as if it was the two-way interaction (part-relation × distinctiveness, which is far from statistical significance). Moreover, the proposed mechanism by which this effect would occur under selection-by-competition is discussed as spreading activation from a target concept to a related distractor (a visible, non-distinctive feature in this case). If this is the mechanism underlying this effect, one should expect no difference between −150 ms and 0 ms SOA: activation of target concept cannot begin before it has appeared. If anything, spread of activation in the other direction [i.e., from feature to its associated concept(s)] should initially drive this effect at −150 ms SOA. And finally, if this effect occurs only when the feature is visible (for counter evidence, see Sailor and Brooks, 2014), we wondered whether there may be a contribution of level of specificity (akin to the basic-level/superordinate naming tasks reviewed by Mahon et al., 2007): might the visibility of the distractor feature permit further activation of its name as a potentially plausible alternative to the basic-level target name?

**Table 1:** **Materials from Vieth et al. (2014) Experiment 2 and 3**.

	**Distinctive**		**Non-distinctive (Exp2)**		**Non-distinctive (Exp3)**	
**Target picture**	**Feature**	**Dominance**	**Feature**	**Dominance**	**Feature**	**Dominance**
BAT	Fangs	7	Stomach	NA		
BED	Springs	7	Foam	NA^a^		
BRA	Hook	9	Cloth	5		
CAMEL	Hump	25	Knee	NA		
CHURCH	Altar	8	Seat	11		
CLOCK	Face	7	Spindle	NA^b^	Glass	NA^d^
COTTAGE	Fireplace	6	Floor	NA		
COW	Udder	8	Liver	NA	Skin	NA
CROCODILE	Jaws	7	Heart	NA	Scales	8
DISHWASHER	Rack	13	Hose	NA	Latch	NA
DUCK	Bill	14	Eye	NA		
ELEPHANT	Trunk	23	Teeth	NA^c^	Toe	NA
ELEVATOR	Cable	9	Ceiling	NA		
GOAT	Beard	14	Tail	6		
GRENADE	Pin	23	Lever	NA		
GUITAR	Hole	8	Fret	NA		
LAMP	Switch	10	Cord	5		
MISSILE	Warhead	6	Engine	NA	Fin	NA
MIXER	Bowl	5	Plug	NA		
MOUSE	Ball	9	Sensor	NA	Button	9
PEACH	Stone	6	Stem	NA		
PIG	Snout	12	Tongue	NA	Hair	NA
PINEAPPLE	Core	6	Stone	NA	Leaf	7
VULTURE	Talons	6	Bone	NA	Wings	8

*The two rightmost columns indicate the non-distinctive features used in Experiment 3 only when they differed from those used in Experiment 2. “NA”: a feature did not appear in McRae et al. (2005) norms, and thus had a dominance of 4 or less in that set*.

a *Most similar feature in McRae et al.'s norms: “has a mattress” (dominance = 18)*.

b *Most similar feature in McRae et al.'s norms: “has hands” (dominance = 18)*.

c *Most similar feature in McRae et al.'s norms: “has tusks” (dominance = 14)*.

d *Most similar feature in McRae et al.'s norms: “has a face” (dominance = 7)*.

Although we appreciate Vieth et al.'s effort to advance our theoretical understanding of lexical retrieval processes through careful manipulation of feature properties, we cannot draw conclusions about the locus of semantic effects in PWI from the present study. Ultimately, a crucial problem is that the details of conceptual representation remain underspecified, and may have major consequences, for example, in predicting whether a feature label should compete with a basic level label (see Vinson et al., 2014). Incorporating explicit models of semantic similarity may offer a way forward in testing current theories of lexical retrieval.

## Conflict of interest statement

The authors declare that the research was conducted in the absence of any commercial or financial relationships that could be construed as a potential conflict of interest.
